# Judgments of Learning Reactivity on Item-Specific and Relational Processing

**DOI:** 10.3390/jintelligence12010004

**Published:** 2024-01-05

**Authors:** Minyu Chang, Charles Brainerd

**Affiliations:** 1 Department of Psychology, McGill University, Montreal, QC H3A 0G4, Canada; 2 Department of Psychology and Human Neuroscience Institute, Cornell University, G331 MVR Hall, Ithaca, NY 14853, USA

**Keywords:** judgment of learning, memory, item-and-relational processing

## Abstract

Judgments of learning (JOLs) reactivity refers to the finding that the mere solicitation of JOLs modifies subsequent memory performance. One theoretical explanation is the item-specific processing hypothesis, which posits that item-level JOLs redound to the benefit of later memory performance because they enhance item-specific processing. The current study was designed to test this account. We factorially manipulated the organization (blocked vs. randomized) of categorized lists and JOL condition (item-JOLs, list-JOLs, no-JOLs) between participants, and fit the dual-retrieval model to free recall data to pinpoint the underlying memory processes that were affected by JOL solicitation. Our results showed that item-level JOLs produced positive reactivity for randomized but not for blocked categorized lists. Moreover, we found that the positive JOL reactivity for randomized categorized lists was tied to a familiarity judgment process that is associated with gist processing, rather than to item-specific recollective processes. Thus, our results pose a challenge to the item-specific processing explanation of JOL reactivity. We argue that JOL reactivity is not restricted to item-specific processing; instead, whether JOLs predominantly engage participants with item-specific or relational processing depends on the interaction between learning stimuli and JOLs.

## 1. Introduction

Accurate metacognitive monitoring is critical for memory performance, and people often regulate their learning strategies based on the self-monitoring of their learning (Dunlosky and Ariel 2011; Kornell and Bjork 2008; Metcalfe and Finn 2008). Judgments of learning (JOLs) are one of the most common measures of people’s metacognitive monitoring, which refers to their predictions of the likelihood of remembering recently encoded material on future memory tests. JOLs were once assumed to assess levels of learning without modifying them. However, a few early researchers questioned this assumption (Nelson and Dunlosky 1992; Spellman and Bjork 1992). More recently, there has been accumulating evidence that JOLs often produce robust learning effects (for reviews, see Double et al. 2018; Double and Birney 2019). The finding that making JOLs directly modifies subsequent memory performance is termed *JOL reactivity*.

Multiple theoretical hypotheses have been proposed to explain JOL reactivity, such as the changed-goal hypothesis (Mitchum et al. 2016), the cue-strengthening hypothesis (Soderstrom et al. 2015), the item-specific processing hypothesis (Senkova and Otani 2021), and the attention-reorienting/enhanced engagement account (Shi et al. 2023; Tauber and Witherby 2019). As background, we provide a summary of these hypotheses in Table 1 and direct readers to other relevant studies that provide support for each theory. It is worth noting here that these theoretical accounts are not mutually exclusive. For instance, the cue-strengthening hypothesis proposes that JOL reactivity results from the strengthening of test-relevant cues, whereas the item-specific processing hypothesis specifies that JOLs’ strengthening effects are tied to item-specific processing. Because those two effects are not logically incompatible, both might occur.

In the current study, we focus on the item-specific processing hypothesis, which evolved from the item-and-relational-processing framework (Einstein and Hunt 1980; Hunt and Einstein 1981). According to this framework, there are two distinct types of processing in list encoding. One is item-specific encoding, which focuses on properties that distinguish individual items from each other (e.g., the unique orthography of a list word). The other is relational encoding, which focuses on properties that different items share (e.g., taxonomic categories and narrative themes). Item-specific and relational processing serve different functions, and memory performance is optimized when both types of processing occur. 

Based on these notions, Senkova and Otani (2021) hypothesized that item-level JOLs resemble encoding tasks that promote item-specific processing. It follows that JOLs should produce larger learning benefits in learning materials that do not normally favor item-specific processing, such as categorized lists, which predominantly trigger relational processing. In support of this hypothesis, Senkova and Otani factorially manipulated JOL condition (JOL, no-JOL) and list type (categorized, unrelated) and found that JOLs enhanced the free recall of categorized lists but not unrelated lists. The hypothesis that making JOLs enhances item-specific processing received further support from the finding that recall for categorized lists following JOLs was comparable to recall following two other encoding manipulations that are known to induce item-specific processing: pleasantness ratings and mental imagery. 

Zhao et al. (2022) also found supporting evidence for the item-specific processing hypothesis using unrelated word lists. They reported that making JOLs improved performance on forced-choice recognition tests and simultaneously impaired performance on order reconstruction tests (i.e., reconstructing the temporal order in which words were encoded). Forced-choice recognition relies heavily on item-specific processing, whereas order reconstruction is an inherently relational task. Thus, the finding that JOLs improve the former and impair the latter suggests that they shift encoding toward item-specific features and away from relational features. Zhao et al. (2023) later replicated the negative effect of JOLs on relational processing with rhyming cue-target word pairs, for which the target words on consecutive pairs were exemplars of the same category. Specifically, they found that making JOLs decreased categorical clustering during the free recall of target words. This again supports the notion that making JOLs slants encoding toward item-specific processing and away from relational processing.

However, there is also evidence that runs counter to the item-specific processing hypothesis. For example, Stevens (2019) found no reactivity of item-level JOLs with Deese–Roediger–McDermott (DRM; Deese 1959; Roediger and McDermott 1995) lists. These lists are composed of words (e.g., *bed*, *pillow*, *yawn*, etc.) that are associated with a common missing word (e.g., *sleep*), which trigger high levels of relational processing. Similarly, Stevens and Pierce (2019) found no reactivity for item-level JOLs with categorized lists, although they did find positive reactivity for *list-level* JOLs. Unlike item-level JOLs, which are made after studying each word, list-level JOLs are made after studying each categorized list. Usually, participants are asked to estimate the number of words they will be able to recall from the list. Stevens and Pierce (2019) argued that item-level JOLs direct participants’ attention to item-specific processing and list-level JOLs direct attention to relational processing. Thus, only list-level JOLs produced positive reactivity whereas item-level JOLs did not because categorical lists prefer relational processing to item-specific processing.

It should be noted that there is a clear discrepancy between Senkova and Otani’s (2021) and Stevens and Pierce’s (2019) findings, as the former authors found positive reactivity of item-level JOLs for categorized lists, whereas the latter did not. In that regard, there is a critical methodological difference between the two studies that may be responsible for the discrepancy. In Stevens and Pierce’s experiments, same-category exemplars were blocked for presentation (i.e., exemplars of the same category are presented consecutively), but in Senkova and Otani’s (2021) experiments, the words’ presentation order was completely randomized. Obviously, the blocked list presentation is more likely to cue relational processing than the randomized presentation.

With this background, the current experiment had two aims. The first was to reconcile the mixed findings of JOL reactivity with categorized lists, and the second was to conduct further tests of Senkova and Otani’s (2021) item-specific processing hypothesis. To achieve the aims, we factorially manipulated the organization of categorized lists (randomized vs. blocked) and JOL condition (item-JOL, list-JOL, and no-JOL), with both variables being manipulated between participants.

Regarding the first aim, we examined whether the two previous findings (positive reactivity vs. no reactivity) could both be obtained in a single experiment with standardized materials and procedures. If so, the two findings are not inconsistent with each other; instead, their discrepancy can be attributed to the level of list organization during encoding. List-level JOLs were also administered as in Stevens and Pierce’s (2019) study. Here, list-level JOLs were expected to produce positive reactivity on recall for blocked but not for randomized categorized lists. This is because, in the randomized list condition, exemplars from different categories are intermixed across lists, leaving no coherent categorical relations within individual lists.

Turning to the second aim, the item-specific hypothesis posits that item-level JOLs improve recall for categorized lists by enhancing item-specific processing, which complements the relational processing that normally predominates. If so, positive reactivity should be observed with both blocked and randomized presentations. In fact, positive reactivity may be stronger with blocked than with randomized presentation, as the former directs attention towards relational processing and away from item-specific processing to a larger extent. 

Moreover, as Senkova and Otani (2021) noted, similar performance between the item-JOL conditions and the pleasantness rating and mental imagery conditions does not guarantee that the underlying processes operating in those conditions are the same or even similar. To address this uncertainty, we used the dual-retrieval model of recall (Brainerd et al. 2009; Chang and Brainerd 2023) to pinpoint the underlying memory processes that are responsible for JOL reactivity in the present experiment. 

The dual-retrieval model was developed based on fuzzy-trace theory’s assumption that people store and retrieve dissociated verbatim and gist traces of experience, that is, the literal traces of individual items versus the traces of semantic, relational, and elaborative information in which items participate (Brainerd and Reyna 1998; Reyna and Brainerd 1995). The only experimental requirement to implement the dual-retrieval model is that participants respond to at least three separate recall tests for encoded items, which supplies sufficient degrees of freedom to estimate all model parameters. There are (a) two types of verbatim retrieval parameters, i.e., direct access (*D*) and forgetting of direct access (*F*), and (b) two types of gist retrieval parameters, i.e., reconstruction (*R*) and familiarity judgment (*J*). The definitions of these parameters can be found in Table 2, and its mathematical machinery is summarized in Appendix A.

Returning to the item-specific processing hypothesis, if JOL reactivity can be accounted for by enhanced item-specific processing, the differences between the item-JOL and no-JOL conditions should be tied to parameters that pertain to item-specific processing, namely, direct access (*D*) or forgetting (*F*) parameters, not parameters that pertain to processing of partial identifying information such as semantic relations across items, namely, reconstruction (*R*) or familiarity judgment (*J*) parameters.

## 2. Materials and Methods

### 2.1. Participants

The participants were 240 young adults (M_age_ = 24.02, SD_age_ = 4.44) recruited from Prolific. They were all fluent English speakers who were residents of the United States, Canada, or the United Kingdom, and they were paid USD 2.33 per person. The participants were randomly assigned to either the item-JOL condition, the list-JOL condition, or the no-JOL condition. Then, within each of those conditions, they were randomly assigned to either a randomized list condition or a blocked list condition. Thus, on average, 40 participants were recruited for each condition, which was comparable to the sample size of Senkova and Otani (2021, Experiment 1). As indicated by Senkova and Otani (2021), a sample size of 26 participants per condition would provide sufficient power (1 − β > 0.80) to detect a medium-sized effect (f = 0.025) based on the power analyses conducted with G*Power (Faul et al. 2007).

### 2.2. Materials

The experiment was programmed and administered via Qualtrics. The study material was 40 single words, which consisted of 8 exemplars from each of 5 taxonomic categories. We used the four categorized lists that were administered by Senkova and Otani (2021) and added another categorized list, which was constructed with the Van Overschelde et al. (2004) category norms (See Appendix B). In the blocked condition, the exemplars of each category were presented consecutively. In the randomized condition, the exemplars of the five categories were randomly mixed and grouped into five new lists, with the constraint that no more than three consecutive words were from the same category.

### 2.3. Procedure

**Figure 1:**
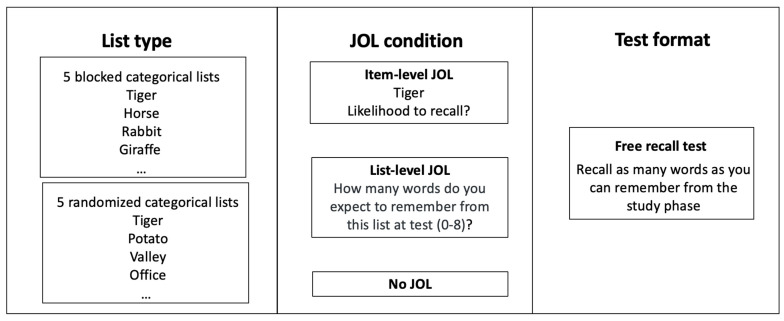
A graphic illustration of the experimental procedure.

A factorial design of 2 (list organization: blocked, randomized) × 3 (JOL condition: item-JOL, list-JOL, no-JOL) was used, where both factors were manipulated between participants. A graphic illustration of the experimental design is shown in Figure 1. All participants completed a study phase and a test phase. In the study phase, the participants encoded 40 words, with each word presented for 2 s. In the item-JOL condition, after each word was presented, it disappeared and a JOL prompt (“Likelihood to recall?”) appeared. Participants were required to rate recall likelihood on a later memory test on a 0–100 scale, with 0 = not likely at all and 100 = totally likely, and they were told to fine-tune their judgments by using the whole 100-point scale. The participants were given a maximum of 4 s to make their JOLs, which they did by typing their responses into a blank box under the JOL prompt. After 4 s, the program automatically proceeded to the next word. In the no-JOL condition, the JOL task was replaced by a random number-generating task as in Senkova and Otani (2021). Specifically, we asked participants to generate a random number between 0 and 100 within 4 s after the presentation of each word. In the list-JOL condition, the participants were also required to generate a random number after each word was studied. In addition, after each list of eight words was presented, the participants were prompted to make a list-level JOL during a 10 s interval between consecutive lists (“How many words do you expect to remember from the list on a later memory test?”). The participants were required to enter a whole number between 0 and 8 into a blank box. During the test phase, the participants completed three consecutive buffer-test cycles. In each cycle, they were first given a 1-minute buffer task (simple arithmetic problems). Then, they were given a maximum of 3 min to recall as many words as possible from the study list. They were told to type the words in any order and that they should not worry about spelling.

## 3. Results

### 3.1. JOL Results

To examine the effects of list organization (blocked vs. randomized) on JOLs, we conducted two separate one-way analyses of variance (ANOVAs) for item-level JOLs and list-level JOLs, respectively. The effects of list organization on item-level JOLs did not reach statistical significance, F(1, 78) = 2.96, MSE = 305.31, η_p_^2^ = 0.04, *p* = .089. In contrast, list-level JOLs were significantly higher for blocked lists (M = 4.89, SD = 1.19) than for randomized lists, (M = 3.73, SD = 1.06), F(1, 79) = 20.89, MSE = 1.29, η_p_^2^ = 0.21, *p* < .001. Thus, list-level JOLs are relatively more sensitive to list organization than item-level JOLs.

### 3.2. Recall Results

**Figure 2:**
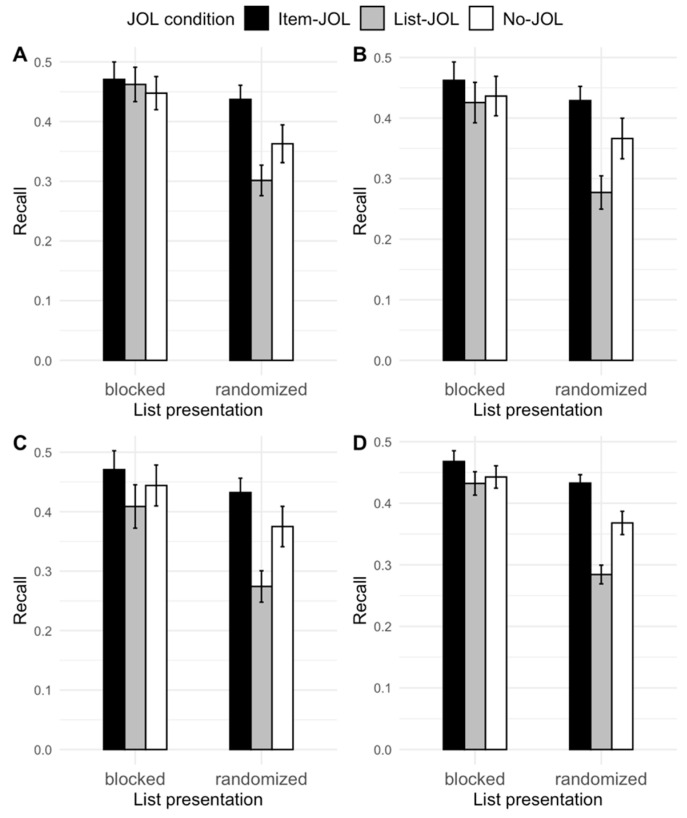
Free recall for blocked categorized lists and randomized categorized lists across the item-, list-, and no-JOL conditions in Experiment 1. Panel (**A**) = recall test 1. Panel (**B**) = recall test 2. Panel (**C**) = recall test 3. Panel (**D**) = average recall across all three tests. Error bars are based on *SE*s.

Three participants’ recall data were identified as outliers as they were 1.5 interquartile ranges (IQRs) above the median (Höhne and Schlosser 2018). These outliers were removed. The removal of the outliers did not change the qualitative effects in the ANOVA results. The recall data are displayed in Figure 2. 

We first conducted 2 (list organization: blocked, randomized) × 3 (JOL condition: item-JOL, list-JOL, no-JOL) × 3 (Test: 1, 2, 3) mixed ANOVA for recall. The ANOVA revealed a main effect of list organization, *F*(1, 231) = 12.71, *MSE* = 0.10, η_p_^2^ = 0.05, *p* < .001, a main effect of JOL condition, *F*(2, 231) = 4.86, *MSE* = 0.10, η_p_^2^ = 0.03, *p* = .009, and a main effect of test, *F*(2, 462) = 6.19, *MSE* = 0.002, η_p_^2^ = 0.03, *p* = .002. Least significant difference (LSD) tests showed that the main effects were due to the fact that recall was higher for blocked lists (*M* = 0.45, *SD* = 0.20) than for randomized lists (*M* = 0.37, S*D* = 0.18), *t(*231) = 3.56, *d* = 0.41, *p* < .001, higher in the item-JOL condition (*M* = 0.45, *SD* = 0.17) than in the list-JOL condition (*M* = 0.37, *SD* = 0.21), *t*(231) = 3.12, *d* = 0.44, *p* = .002, and higher on the first recall test (*M* = 0.42, *SD* = 0.18) than on the second (*M* = 0.40, *SD* = 0.20), and the third recall test (*M* = 0.40, *SD* = 0.21), *t*(231) = 3.13 and 2.45, *d*s = 0.20 and 0.16, *p*s < .015. Additionally, there was a JOL condition × Test interaction, *F*(4, 468) = 2.99, MSE = 0.004, η_p_^2^ = 0.03, *p* = .019. The JOL condition effect was significant across all three recall tests, *p*s < .032, but it increased slightly from test 1 to test 2 to test 3. Last, the interaction that is of primary interest, the JOL condition × List organization interaction, was not significant, *F*(2, 231) = 1.87, *MSE* = 0.10, η_p_^2^ = 0.02, *p* = .156.

Although the JOL condition × List organization interaction did not reach the conventional criterion of statistical significance, we conducted a planned one-way ANOVA to compare recall between the item-JOL, list-JOL, and no-JOL conditions for randomized lists. Considering that we found significant differences in recall between test 1 and the following tests and that Senkova and Otani (2021) only administered a single study-test cycle, we only included test 1 data to make this analysis comparable to that of Senkova and Otani (2021). The one-way ANOVA showed that the effect of JOL condition was significant, *F*(2, 114) = 6.52, *MSE* = 0.03, η_p_^2^ = 0.10, *p* = .002. LSD tests indicated that the item-JOL condition (*M* = 0.44, *SD* = 0.16) produced a higher recall for randomized lists than both the list-JOL condition, (*M* = 0.30, *SD* = 0.15), *t*(114) = 3.58, *d* = 0.88, *p* < .001, and the no-JOL condition, (*M* = 0.36, *SD* = 0.19), *t*(114) = 1.99, *d* = 0.42, *p* = .049. Therefore, we replicated Senkova and Otani’s result that the item-JOLs produced a better recall for randomized categorized lists compared to that of the no-JOL condition. 

Additionally, we conducted another planned one-way ANOVA to compare recall between item-JOL, list-JOL, and no-JOL conditions for blocked lists. We restricted this analysis to test 1 data for the same reason stated above. The ANOVA showed that there was no difference in recall for blocked lists between the item-JOL (*M* = 0.47, *SD* = 0.17), list-JOL (*M* = 0.46, *SD* = 0.19), and no-JOL conditions (*M* = 0.45, *SD* = 0.18), F(2, 117) = 0.16, *MSE* = 0.03, η_p_^2^ = 0.003, *p* = .851. Therefore, we reproduced Stevens and Pierce’s (2019) finding of null reactivity of item-level JOLs for blocked categorized lists. In brief, we resolved the inconsistency between Senkova and Otani’s (2021) and Stevens and Pierce’s (2019) results by demonstrating that it is tied to whether the presentation of categorized lists is blocked or randomized.

### 3.3. Model Results

The free recall data were further analyzed with the dual-retrieval model. As can be seen in Table 3, the average *G*^2^(1) across all possible combinations between JOL conditions (item-, list-, and no-JOL) and list organization (blocked and randomized) is 3.56. Because *G*^2^(1) is asymptotically distributed as *χ*^2^(1), the goodness of fit is evaluated by comparing the observed *G*^2^(1) to the critical value of *χ*^2^(1) for rejecting the null hypothesis, which is 3.84 at the 0.05 confidence level. Thus, the observed fit level was acceptable. 

For the blocked lists, the comparisons of primary interest are between the item-JOL and no-JOL conditions. There were no significant differences in any model parameter between those two conditions, which is consistent with the ANOVA results. Next, we consider the comparisons between the list-JOL conditions and the other two JOL conditions. The *F* parameter was larger in the list-JOL condition (0.10) than in the item-JOL and no-JOL conditions (0.05 and 0.05), ∆*G*^2^s > 15.38, *p*s < .001. Furthermore, the *J*_2_ parameter was larger in the list-JOL condition than in the item-JOL condition, ∆*G*^2^(1) = 4.58, *p* = .032. This suggests that list-level JOLs simulated more forgetting of item-specific verbatim details and that words followed by list-level JOLs felt more familiar on the later recall tests relative to those followed by item-level JOLs.

The patterns were quite different for randomized lists. We first consider the comparison between the item-JOL and no-JOL conditions. Here, the *J*_1_ parameter was larger in the item-JOL condition (0.54) than in the no-JOL condition (0.34), ∆*G*^2^(1) = 4.56, *p* = .033, suggesting that item-JOLs increased familiarity for reconstructed words. No other condition-wise difference in parameters reached statistical significance.

Next, we examine parameter differences between the list-JOL condition and the other two JOL conditions. Here, the *D* parameter was smaller in the list-JOL condition (0.25) relative to the item-JOL condition (0.37) and the no-JOL condition (0.33), ∆*G*^2^s > 11.87, *p*s ≤ .001, and the *F* parameter was again larger in the list-JOL condition than in the item-JOL condition (0.09 vs. 0.06), ∆*G*^2^(1) = 3.89, *p* = .046. This suggests that list-level JOLs impaired initial verbatim retrieval and increased its forgetting. Meanwhile, the *J*_1_ parameter was larger in the list-JOL condition (0.71) than in the item-JOL condition (0.54) or the no-JOL condition (0.43), ∆*G*^2^s > 8.34, *p*s < 0.004. Last, the *R* parameter was smaller in the list-JOL condition than in the item-JOL condition (0.20 vs. 0.10), ∆*G*^2^(1) = 5.20, *p* = .023. Thus, at the level of underlying memory processes, the list-JOL condition impaired both verbatim retrieval and reconstruction, but it made items seem more familiar during recall.

### 3.4. Follow-Up Analysis

The analyses so far show that item-level JOLs produced positive reactivity for randomized lists but not for blocked lists. Additionally, the positive reactivity of item-level JOLs was localized within familiarity judgment (*J*_1_) rather than direct access (*D*) or forgetting (*F*). Together, these results are not congruent with the notion that item-level JOLs improve the recall of randomized lists by enhancing item-specific processing. Instead, it appears that item-level JOLs may have enhanced relational processing, by increasing meta-cognitive awareness that a randomly ordered series of words can be grouped into categories. 

To test this alternative hypothesis, we conducted a follow-up one-tailed *t*-test that compared category clustering between the item-JOL and no-JOL conditions for the randomized lists. According to the item-and-relational-processing framework (Einstein and Hunt 1980; Hunt and Einstein 1981), relational processing should enhance category clustering during recall, but item-specific processing should not. We used the adjusted ratio of clustering (ARC; Roenker et al. 1971) as the index of category clustering, with 0 indicating chance clustering and 1 indicating perfect clustering. ARC is calculated as follows:

$$\mathrm{ARC}=\left\{\begin{array}{c} \frac{R-E(R)\;}{maxR-E(R)},\;\;if\;R>E(R)\\ \frac{R-E(R)\;}{E\left(R\right)-minR\;},\;if\;R<E(R) \end{array}\right.$$

Here, R  is the total number of category repetitions (i.e., situations where a category exemplar follows another exemplar from the same category), $E\left(R\right)\;$ is the expected number of category repetitions by chance, maxR is the maximum possible number of category repetitions, and minR is the minimum possible number of category repetitions. $E\left(R\right)$, maxR, and minR are calculated as follows:

$$E\left(R\right)\;=\frac{\sum_{i}n_{i}^{2}}{N}-1$$

maxR=N−k

$$minR=\left\{\begin{array}{cc} 0, & if\;N+1\geq 2m\\ 2m-N-1, & otherwise \end{array}\right.$$

where $n_{i}$ is the number of items recalled from the category *i*, *N* is the total number of items recalled, *k* is the number of categories to which recalled items belong, and *m* is the number of items in the category with the most items recalled.

Again, we confined our analyses to test 1 for randomized lists. The *t*-test showed that the difference in category clustering between the item-JOL condition (*M* = 0.35, *SD* = 0.36) and the no-JOL condition (*M* = 0.19, *SD* = 0.56) approached but did not reach significance, *t*(79) = 1.53, *d* = 0.34, *p* = .065. However, it is worth mentioning that we did not have sufficient statistical power for the follow-up analysis, as post hoc power analyses showed that with a *df* of 79, and a small effect size of *d* = 0.34, we only had a power of 0.45 to detect a significant effect in the independent *t*-test.

## 4. Discussion

In the current study, supporting evidence was found for our explanation of the discrepant findings of Stevens and Pierce (2019) versus Senkova and Otani (2021): We found that the reactivity of item-level JOLs for categorized lists is controlled by list organization. Item-level JOLs produced positive reactivity when list words were randomized but not when they were blocked by category. Moreover, the dual-retrieval model revealed that the recall advantage for randomized lists in the item-JOL condition was driven by the enhancement in gist parameters rather than verbatim parameters. More specifically, making JOLs did not improve retrieval of item-specific verbatim traces, but it increased the feelings of familiarity with words that the participants had reconstructed based on gist traces. 

The finding that item-level JOLs enhanced recall for randomized but not for blocked categorized lists cannot be accommodated by Senkova and Otani’s (2021) item-specific processing hypothesis. According to this hypothesis, categorized lists encourage relational processing, whereas unrelated lists promote item-specific processing. Consequently, if item-level JOLs enhance item-specific processing, they should improve memory for categorized lists more than memory for uncategorized lists, because encoding is largely shifted toward relational features and away from item-specific features in the former. However, the hypothesis expects positive JOL reactivity for categorized lists regardless of whether they are randomized or blocked. Actually, it expects stronger positive reactivity for blocked lists owing to the dominance of relational processing with blocked lists (Ackerman 1986). 

Then, why did the reactivity of item-level JOLs only occur in randomized but not blocked lists? One possible explanation offered by the model analysis is that positive JOL reactivity for randomized lists mainly results from enhanced familiarity of items that are reconstructed from partial-identifying gist traces. As can be seen in Table 3, item-level JOLs improved familiarity judgment (*J*_1_) but not item-specific recollection (*D*) for randomized lists, whereas they only affected verbatim forgetting (*F*) for blocked lists. Thus, it is possible that the improvement in relational processing was a key determinant of positive reactivity of item-level JOLs in categorized lists, that is, making JOLs may heighten the awareness that individual words can be grouped together under specific categories. If that is the case, it is obvious that enhancement of relational processing should be more beneficial for randomized than for blocked lists. With blocked lists, high levels of relational processing would spontaneously be afforded, while with randomized lists, the exemplars of the same category are randomly scattered around, which hinders relational processing relative to blocked lists. Therefore, if positive reactivity of item-level JOLs for categorized lists is driven by relational processing, such benefits will be relatively redundant for blocked lists but complementary for randomized lists. However, it must be acknowledged that this explanation is speculative because our experiment was not designed to test it. We did conduct follow-up clustering analyses to test it, which showed that item-level JOLs enhanced category clustering for randomized lists at the trend level. Given that the follow-up analysis was underpowered, we recommend that this result ought to be further replicated and examined in future research.

In brief, our findings about categorized lists pose challenges to the item-specific processing hypothesis because (a) we observed positive JOL reactivity only for randomized lists but not blocked lists, and (b) positive JOL reactivity for randomized lists was localized within retrieval processes that index relational processing. Although the item-specific processing hypothesis cannot accommodate the current data, it did provide a good account of JOL reactivity data in other studies. For example, in Experiments 1 and 2 of Chang and Brainerd (2023), it was observed that positive JOL reactivity for related word pairs was tied to dual-retrieval model parameters that index item-specific recollection. Moreover, according to Zhao et al. (2022, 2023), item-level JOLs disrupt order reconstruction (a measure of relational processing) with unrelated word lists and rhyming pairs whose target words are categorical exemplars. However, it is worth pointing out that those stimuli (word pairs and unrelated lists) naturally trigger greater levels of item-specific processing as compared to the categorized lists we used. Therefore, the types of processing (item-specific or relational) that are enhanced by JOLs may depend heavily on the characteristics of the items that people encode. 

Last, we had expected that list-level JOLs would not produce reactivity for randomized lists because list-level JOLs direct participants’ attention to the relations among words on the same list when these words are not meaningfully related. Our results were consistent with this prediction. Additionally, the experiment showed that neither item- nor list-level JOLs enhanced the recall of blocked lists. The former finding is consistent with Stevens and Pierce’s (2019) results, whereas the latter is not. A possible reason why list-level JOL reactivity for blocked lists was not replicated is the difference in test format: Stevens and Pierce used a cued recall procedure that provided category labels as retrieval cues, whereas we used free recall. If list-level JOLs slant encoding toward relational processing, cued recall, which facilitates relational processing, should be more sensitive to JOL reactivity than free recall.

In summary, our results showed that JOL reactivity is a contextual memory effect that depends heavily on the interactions between learning material, JOL type, and memory test format. This notion echoes the cue-strengthening hypothesis, whose main assumption is that JOL reactivity depends on whether JOLs strengthen the cues afforded by the learning stimuli and what type of cues are favored by the memory tests. In addition, our argument is also highly consistent with the tetrahedral model of memory (Jenkins 1979). The model posits that memory effect is dependent on four dimensions: subject characteristic (e.g., ability), learning stimuli (e.g., type of learning material), encoding task (e.g., instructions provided at encoding), and memory test (e.g., recall, recognition, etcs), and moreover, the interactions between them. Our findings tap the interactions between the last three dimensions in the model. Regarding the interaction between learning stimuli and encoding task, for learning materials where exemplars under the same category are scattered around (randomized) rather than blocked together, only item-JOLs pick up and strengthen the interitem relational cue. On the contrary, list-JOLs would be misleading for randomized lists as they slant participants’ attention to interitem relation among consecutive words, while they are not meaningfully related. Moreover, whether the cue strengthening eventually transfers to positive reactivity also depends on the sensitivity of memory tests to the strengthened cues. This may explain why list-JOL reactivity was observed for blocked lists with cued recall but not free recall.

## 5. Conclusions

The current study identifies a boundary condition that explains prior mixed findings about JOL reactivity with categorized lists, namely, reactivity depends on the level of list organization during encoding. More importantly, this finding poses challenges to the item-specific processing hypothesis, which predicts positive reactivity for both blocked and randomized categorized lists. We observed that positive JOL reactivity is primarily localized within retrieval operations that involve relational rather than item-specific processing, in contrast to the prediction of the item-specific processing hypothesis. We argue that enhanced item-specific processing cannot fully account for JOL reactivity and that the specific processes that are strengthened by JOLs depend heavily on the interaction between learning stimuli and JOLs. Our results are conceptually consistent with the cue-strengthening hypothesis and the tetrahedral model of memory.

## Figures and Tables

**Table 1:** Summary of theoretical accounts of JOL reactivity.

Theory	Explanations	Major Studies Supporting the Theories
Changed-goal hypothesis	Making JOLs heightens participants’ awareness of item differences in learning difficulty, thus prompting them to emphasize easier items (e.g., related word pairs) at the expense of harder items (e.g., unrelated word pairs).	(Janes et al. 2018; Mitchum et al. 2016; Schäfer and Undorf 2021)
Cue-strengthening hypothesis	Making JOLs strengthens the cues that inform JOLs (e.g., cue-target relatedness in related word pairs), and thus produces memory benefits when future memory tests are sensitive to those cues.	(Chang and Brainerd 2023; Halamish and Undorf 2022; Maxwell and Huff 2023; Myers et al. 2020; Rivers et al. 2021, 2023; Soderstrom et al. 2015; Tekin and Roediger 2020; Witherby and Tauber 2017)
Item-specific processing hypothesis	Making JOLs promotes item-specific processing and thus enhances the distinctiveness of individual items.	(Senkova and Otani 2021; Zhao et al. 2022, 2023)
Attention-reorienting account/enhanced engagement account	Making JOLs enhances the attention to the study items and increases learners’ engagement in processing the items.	(Shi et al. 2023; Tauber and Witherby 2019; Murphy et al. 2023)

**Table 2:** Definitions of parameters in the dual-retrieval model.

Parameters	Definitions
*D*	Direct access/recollection: the probability that the verbatim traces of an item’s prior presentation can be retrieved on a recall test.
*F*	Forgetting of direct access: the probability that direct access to an item’s verbatim traces is lost after the first or second recall test due to forgetting.
*R*	Reconstruction: the probability that when direct access fails, an item can be reconstructed on a recall test using gist traces (e.g., inter-item semantic relation).
*J*_1_, *J*_2_, *J*_3_	Familiarity judgment: The probability that a reconstructed item is judged to be familiar enough to output. *J*_1_, *J*_2_, and *J*_3_ represent the familiarity judgments for the first, second, and third recall tests, respectively.

**Table 3:** Dual-retrieval model fits and parameter estimates for Experiment 1.

List Organization	JOL Condition	*G* ^2^	*D*	*F*	*J*_1_	*J*_2_	*J*_3_	*R*
Blocked								
	Item-JOL	0.00	0.42	**0.05**	0.40	**0.52**	0.79	0.19
	List-JOL	13.91	0.42	**0.10**	0.50	**0.69**	0.78	0.20
	No-JOL	2.69	0.40	**0.05**	0.42	0.62	0.84	0.20
Randomized								
	Item-JOL	0.31	**0.37**	**0.06**	**0.54**	0.64	0.84	**0.20**
	List-JOL	4.34	**0.25**	**0.09**	**0.71**	0.74	0.91	**0.10**
	No-JOL	0.11	**0.33**	0.06	**0.34**	0.58	0.84	0.15

*Note: D* = direct access parameter; *F* = forgetting parameter; *J*_1_ = familiarity judgment parameter for test 1; *J*_2_ = familiarity judgment parameter for test 2; *J*_3_ = familiarity judgment parameter for test 3; and *R* = reconstruction parameter. Parameters that differed reliably between JOL conditions are printed in boldface.

## Data Availability

All raw data have been uploaded to the Open Science Framework and are available at https://osf.io/csu5d/.

## Appendix A

The dual-retrieval model used in the current experiments is described below:

p(CCC) = *D*(1 − *F*)(1 − *F*) + (1 − *D*)*RJ*_1_*J*_2_*J*_3_ (A1)

p(CCE) = *D*(1 − *F*)*F* + (1 − *D*)*RJ*_1_*J*_2_(1 − *J*_3_) (A2)

p(CEC) = (1 − *D*)*RJ*_1_(1 − *J*_2_)*J*_3_ (A3)

p(CEE) = *DF* + (1 − *D*)*RJ*_1_(1 − *J*_2_)(1 − *J*_3_) (A4)

p(ECC) = (1 − *D*)*R*(1 − *J*_1_)*J*_2_*J*_3_ (A5)

p(ECE) = (1 − *D*)*R*(1 − *J*_1_)*J*_2_(1 − *J*_3_) (A6)

p(EEC) = (1 − *D*)*R*(1 − *J*_1_)(1 − *J*_2_)*J*_3_ (A7)

p(EEE) = (1 − *D*)*R*(1 − *J*_1_)(1 − *J*_2_)(1 − *J*_3_) + (1 − *D*)(1 − *R*) (A8)

where *D* is the probability that the verbatim trace of an item’s presentation can be directly accessed on a recall test, *F* is the probability that the direct access works in the first recall test but fails in the second or third recall test, *R* is the probability that an item can be reconstructed on a recall test when the verbatim trace of the item’s presentation cannot be accessed, and *J*_1_*, J*_2_*,* and *J*_3_ are the probabilities that a reconstructed item is judged to be familiar enough to output on test 1, test 2, and test 3, respectively.

It should be noted that the current version of the dual-retrieval model is slightly different from the prior version used in Chang and Brainerd (2023). The prior version applied to associative recall of word pairs, whereas the current model was developed specifically for free recall tests for lists of single words. The only difference between the two versions is that the prior version assumes that the forgetting status remains invariant between the second and third recall tests, but the current version can cover the situation that participants retained direct access in the second recall test but forgot it in the third recall test. For instance, the *F* parameter no longer stands for the forgetting probability in both recall tests 2 *and* 3 in the prior version. Instead, it is now defined as the probability that participants lost direct access due to forgetting in the second *or* the third recall test, with the assumption that the probability of forgetting was equal between the two recall tests.

The likelihood function for the data predicted by the dual-retrieval model is as follows:

*L*_6_ = Π(*p_i_*)*^N^*^(*i*)^ (A9)

where *p_i_* is the predicted recall probabilities on the left side of all the aforementioned equations, and the *N*_(*i*)_ is actual observed data counts. Because six parameter estimates are obtained with the model, one empirical probability is free to vary, namely, there is one degree of freedom for *L*_6_.

To estimate goodness of fits, the likelihood in Equation (A9) was compared to the likelihood of the same data when all empirical probabilities are free to vary. The goodness-of-fit test is as follows:

*G*^2^ = −2ln[*L*_6_/*L*_7_] (A10)

where *L*_6_ is the likelihood of the data predicted by the dual-retrieval model, and *L*_7_ is the likelihood of the same data when all empirical probabilities are free to vary*. G*^2^ has a similar asymptotic distribution as χ^2^. Thus, the critical value of rejecting the null hypothesis at the 0.05 confidence level is 3.84.

## Appendix B

Blocked lists:

**Categorical Label**	**A Natural Earth Formation**	**A Vegetable**	**A Four-Footed Animal**	**A Part of a Building**	**A Musical Instrument**
List words	valley	potato	tiger	office	drum
	river	squash	horse	stairs	guitar
	canyon	pepper	rabbit	lobby	flute
	volcano	lettuce	giraffe	ceiling	piano
	ocean	radish	elephant	window	trumpet
	cliff	carrot	moose	elevator	clarinet
	island	tomato	squirrel	basement	violin
	stream	cabbage	raccoon	floor	cello

Randomized lists:

**List Label**	**List 1**	**List 2**	**List 3**	**List 4**	**List 5**
List words	lettuce	squash	valley	drum	ocean
	river	rabbit	giraffe	tomato	tiger
	trumpet	pepper	guitar	basement	radish
	ceiling	elevator	flute	squirrel	office
	moose	elephant	carrot	cabbage	cello
	canyon	piano	volcano	horse	stream
	raccoon	lobby	island	floor	clarinet
	stairs	potato	violin	cliff	window
